# Whole genome sequencing across clinical trials identifies rare coding variants in *GPR68* associated with chemotherapy-induced peripheral neuropathy

**DOI:** 10.1186/s13073-023-01193-4

**Published:** 2023-06-21

**Authors:** Zia Khan, Min Jung, Megan Crow, Rajat Mohindra, Vidya Maiya, Joshua S. Kaminker, David H. Hackos, G. Scott Chandler, Mark I. McCarthy, Tushar Bhangale

**Affiliations:** 1 Genentech, 1 DNA Way, South San Francisco, 94080 USA; 2 F. Hoffmann-La Roche, Grenzacherstrasse 124, 4070 Basel, Switzerland

**Keywords:** Chemotherapy-induced peripheral neuropathy, CIPN, GPR68, GRID2, Cancer, Whole genome sequencing, Neurological toxicity, Rare coding variants, Pharmacogenomics, Taxane, Paclitaxel

## Abstract

**Background:**

Dose-limiting toxicities significantly impact the benefit/risk profile of many drugs. Whole genome sequencing (WGS) in patients receiving drugs with dose-limiting toxicities can identify therapeutic hypotheses to prevent these toxicities. Chemotherapy-induced peripheral neuropathy (CIPN) is a common dose-limiting neurological toxicity of chemotherapies with no effective approach for prevention.

**Methods:**

We conducted a genetic study of time-to-first peripheral neuropathy event using 30× germline WGS data from whole blood samples from 4900 European-ancestry cancer patients in 14 randomized controlled trials. A substantial number of patients in these trials received taxane and platinum-based chemotherapies as part of their treatment regimen, either standard of care or in combination with the PD-L1 inhibitor atezolizumab. The trials spanned several cancers including renal cell carcinoma, triple negative breast cancer, non-small cell lung cancer, small cell lung cancer, bladder cancer, ovarian cancer, and melanoma.

**Results:**

We identified a locus consisting of low-frequency variants in intron 13 of *GRID2* associated with time-to-onset of first peripheral neuropathy (PN) indexed by rs17020773 (*p* = 2.03 × 10^−8^, all patients, *p* = 6.36 × 10^−9^, taxane treated). Gene-level burden analysis identified rare coding variants associated with increased PN risk in the C-terminus of *GPR68* (*p* = 1.59 × 10^−6^, all patients, *p* = 3.47 × 10^−8^, taxane treated), a pH-sensitive G-protein coupled receptor (GPCR). The variants driving this signal were found to alter predicted arrestin binding motifs in the C-terminus of *GPR68*. Analysis of snRNA-seq from human dorsal root ganglia (DRG) indicated that expression of *GPR68* was highest in mechano-thermo-sensitive nociceptors.

**Conclusions:**

Our genetic study provides insight into the impact of low-frequency and rare coding genetic variation on PN risk and suggests that further study of *GPR68* in sensory neurons may yield a therapeutic hypothesis for prevention of CIPN.

**Supplementary Information:**

The online version contains supplementary material available at 10.1186/s13073-023-01193-4.

## Background

A strategy to prevent a dose-limiting toxicity of a drug can enhance benefit/risk balance to enable more optimal dosing, tolerability, and ultimately improved patient outcomes. This is particularly true in chemotherapies where dose-density is correlated with benefit and dose-limiting toxicities can prevent patients from achieving the desired disease control. Yet, identifying such a therapeutic strategy is challenging as models of these toxicities in animals and in organoid systems may not accurately reflect events in patients [1]. Patients differ in their tendency to develop dose-limiting toxicities and vary in the severity of toxicities they experience suggesting that genetic variation is a contributing factor [2]. Germline WGS in these patients can elucidate the genetic architecture of toxicity risk and identify rare coding variants associated with this risk. This approach has the potential to yield biological insights that can lead to therapeutic approaches for toxicity prevention and drug combinations that provide patients with a more favorable benefit/risk profile.

Peripheral neuropathy (PN) is a common dose-limiting, cumulative neurological toxicity of chemotherapy. PN often begins with sensory deficits and paresthesia in the hands and feet, following a “glove and stocking” distribution due to damage to longer neurons [3]. Painful sensations and allodynia are also common. In severe cases, PN can persist after the cessation of cancer therapy. Despite several decades of study, the prevention or treatment of chemotherapy-induced PN (CIPN) remains challenging [4]. Numerous genome-wide association studies (GWAS) and candidate gene studies have been conducted using array based genotyping to identify variants associated with CIPN [5–9] (reviewed in [10]). Yet, these prior studies have been limited to a small number of cohorts and cancer indications—mainly due to the challenges in uniformly grading the PN events and compiling harmonized clinical or phenotype data at scale. Moreover, the impact of low-frequency or rare genetic variation on PN has not been examined.

We conducted a GWAS of time-to-first PN event using whole genome sequencing data collected from whole blood samples of European ancestry cancer patients from 14 previously completed randomized controlled trials. We identified one locus, indexed by rs17020773, on chromosome 4 in intron 13 of *GRID2* reaching genome-wide significance for earlier time to onset of CIPN, a signal driven by patients receiving taxane-based chemotherapies. Using rare coding variant burden analysis, we also identified variants in *GPR68*, a pH-sensitive GPCR, that were associated with risk of earlier onset of CIPN. Within human DRG snRNA-seq, we found that expression of *GPR68* was highest in PEP1 sensory neurons which are mechano-thermo-sensitive nociceptors. Our study provides insight into the genetic etiology of CIPN and human genetic evidence that supports further study of the role of both *GRID2* and *GPR68* in CIPN risk and prevention.

## Methods

### Patient cohort

A retrospective genetic study of peripheral neuropathy events was conducted using individual participant data from 14 previously completed randomized controlled trials. Detailed clinical trial results have been previously reported for IMmotion151 [11], IMpassion130 [12], IMpower110 [13]/130 [14]/131 [15]/132 [16]/150 [17], IMpower133 [18], IMvigor010 [19]/130 [20]/211 [21], IMagyn050 [22], IMspire170 [23], and GO29779 [24]. The original publications provide detailed protocols and inclusion criteria for the clinical trials.

As these trials were enrolled, a subset of patients signed an optional Research Biosample Repository (RBR) Informed Consent Form (ICF) to provide whole blood samples for future research. By signing the optional RBR ICF, patients provided informed consent for study of inherited and non-inherited genetic variation from these whole blood samples. Between the years 2016 and 2021, whole genome sequencing data was collected from whole blood only from patients that signed the optional RBR ICF by the Human Genetics Initiative at Genentech.

Peripheral neuropathy event data and all meta-data were harmonized from these studies for 4900 patients with more than 70% European ancestry and whose whole genome sequencing data met all QC criteria (see below). The number of patients analyzed from each clinical trial is summarized in Additional file 1: Table S1. This individual participant data was then used for the single variant GWAS and for rare coding variant burden testing.

### Whole genome sequencing

Genomic DNA was extracted from whole blood samples using the DNA Blood400 kit (Chemagic) and eluted in 50μL Elution Buffer (EB, Qiagen). DNA was sheared (Covaris LE220) and sequencing libraries were prepared using the TruSeq Nano DNA HT kit (Illumina Inc.). Libraries were sequenced at Human Longevity (San Diego, CA, USA) and the Broad Institute (Boston, MA, USA). All sequencing data was checked for concordance with SNP fingerprint data collected before sequencing. 150 bp paired-end whole genome sequencing (WGS) data was generated to an average read depth of 30× using the HiSeq platform (Illumina X10, San Diego, CA, USA).

### Sample and variant level QC

Reads were aligned using the functionally equivalent (FEB) BAM pipeline [25]. Samples were jointly genotyped using Sentieon version of GATK (Sentieon Inc.). Only variants flagged as PASS and genotype calls with GQ > 20 were used. After application of the GQ filter, variants with genotype call missing rate of > 0.01 were removed. Multi-allelic sites were handled by variant splitting using bcftools [26]. Samples were removed if the proportion of sites with missing genotypes exceeded 0.1. Variants with minor allele frequency (MAF) > 0.01 were extracted, and LD-based pruning using PLINK was performed, prior to merging with 1000 Genomes data. Then, ADMIXTURE v1.23 was used in supervised mode to estimate ancestry along the five major populations defined in the 1000 Genomes data [27, 28]. Only samples with European ancestry EUR > 0.7 were used in subsequent steps. Observed and expected homozygous/heterozygous genotype counts for each sample were tabulated and method-of-moments estimates of the *F*-coefficient were generated for each sample. Samples with an *F*-statistic more than five standard deviations above the mean were removed. Samples were then analyzed for relatedness using the KING-robust method as implemented in plink2 [29]. Sample pairs with a KING kinship coefficient greater than 0.177 were identified, and one of the pair of samples was removed randomly. We then performed PCA using the implementation in the proPCA package [30]. Six rounds of PCA outlier removal iterations were performed. Samples that were > 5 standard deviations from the top five eigenvectors at each iteration were removed from the analysis. The final PCA was then performed to compute five eigenvectors that were subsequently used to account for any remaining population stratification. Variants were also analyzed for violation of Hardy–Weinberg equilibrium (HWE) and those with HWE *p*-value below 10^−8^ were excluded from the analysis. Variants with MAF > 0.01 were designated as common variants. Missense variants were annotated using bcftools csq [31]. We used missense variants associated with canonical coding transcript for a gene designated in Ensembl Release-104 of the genome build GRCh38.p13. In total, there were 605,052 missense variants found in our cohort at all allele frequencies.

### Identification of PN events

Pre-existing grade ≥ 2 peripheral neuropathy as defined by National Cancer Institute Common Terminology Criteria for Adverse Events (NCI CTCAE) was an exclusion criterion for trials where chemotherapy was used. PN events were diagnosed by study investigators in accordance with standard of care, institutional practice, and the trial protocols. The protocols indicated that a consistent methodology of non-directive questioning should be adopted for eliciting adverse event information at all patient evaluation time points. Examples of non-directive questions include the following: “How have you felt since your last clinic visit?” “Have you had any new or changed health problems since you were last here?” Investigators were instructed to use correct medical terminology/concepts when recording adverse events on the adverse event electronic case report form (eCRF) and to record only one adverse event term in the event field on the adverse event eCRF. Investigators were asked to grade the event according to the NCI CTCAE criteria whereby grade 2 or higher PN events were distinguished on the basis of their impact on ADL. Patient-reported outcomes (PROs) were excluded from adverse event reporting. The medical concepts in the Adverse Event eCRF were mapped by a consistent methodology to MedDRA terms. All events under MedDRA:10,034,607 Peripheral neuropathies NEC were used as PN events within our study. PN events of all grades were used in our study.

Time-to-event data started at time from first treatment. This continues through the follow-up time of the patient to censoring or the PN event. The adverse event (AE) reporting window for all adverse events is 30 days after the final dose or until initiation of a new systemic anti-cancer therapy, whichever comes first. For serious adverse events or adverse events of special interest, the AE reporting window is 90 days after the final dose or until initiation of new therapy, whichever occurs first. After the adverse event reporting period, if the study investigator becomes aware of any serious events believed to be related to the therapies tested in the trial arm, this information is reported. All but 3 trials required patients to be chemotherapy naive as the majority of the trials were conducted in the first line setting. The only exceptions were IMvigor211, IMvigor010, and the IDO-inhibitor trial. IMvigor211 was a trial in the second line setting where patients progressed during or following a platinum-containing regimen. IMvigor010 allowed patients that received prior neoadjuvant chemotherapy. The IDO-inhibitor trial allowed patients to have received prior lines of therapy of which could include chemotherapies. All the patients in the taxane-treated subcohort did not previously receive taxanes in any line of therapy.

### Time-to-event GWAS

We found that the baseline hazard for time-to-first PN differed in each trial arm. The incidence plots or event-free Kaplan–Meier curves for time-to-PN differed between trial arms and could cross to violate the proportional hazard’s assumption of a Cox model. In a meta-analysis, this could be addressed by fitting a Cox model separately in each trial arm and combining the coefficient associated with the covariate of interest (e.g. genotype) by inverse variance weighting. However, in some of the trial arms, PN events were rare, and the overall number of patients in the trial arm was small which makes it difficult to obtain an accurate fit of a Cox model as the likelihood is not well specified. Since we had individual participant data in our study, inverse variance weighting was not a requirement. We therefore used an approach also known as one-stage meta-analysis using individual participant data [32]. By stratifying the Cox model by trial arm, this approach accounts for the different baseline hazard for PN in each trial arm. As the likelihood is well specified, this approach also accounts for both rare events and small trial arms.

The GWAS was adjusted for five eigenvectors from genotype PCA, sex, and age. We stratified the model by trial arms to allow us to conduct an individual participant data meta-analysis. The final Cox model could be specified using the coxph function in the R survival package as follows: coxph(Surv(PN.time, PN.occured) ~ dosage + EV.1 + EV.2 + EV.3 + EV.4 + EV.5 + AGE + SEX + strata(trial.arm)). The *p*-value corresponded to a two-sided Wald-test for a non-zero coefficient on the dosage term in the model.

### Time to event rare variant burden tests

We conducted burden tests using rare coding variants (MAF < 0.01). We limited these rare variant burden tests to genes with at least 30 rare coding variant carriers across the cohort. We tested for the association between the number of rare variants carried by an individual (burden) with the time to a PN event. We note that the burden test assumes the effects of rare variants have the same direction. The Cox model used can specified using the coxph function the R survival package as follows: coxph(Surv(PN.time, PN.occured) ~ burden + EV.1 + EV.2 + EV.3 + EV.4 + EV.5 + AGE + SEX + strata(trial.arm)). The burden test *p*-value corresponded to a two-sided Wald-test for a non-zero coefficient on the dosage term in the model.

### Polygenic risk score analyses

We constructed PRS models using genome-wide summary statistics from two studies: (1) GWAS of hereditary neuropathy derived from individuals in the UK Biobank (GWAS catalog GCST90038643) [33] and (2) time-to-PN analysis in all patients from this study. Both models were generated using PRS-CS with the default set of parameters and UK Biobank LD reference panel for European ancestry provided by PRS-CS authors with the default set of parameters [34]. LD-pruned PRS were computed using PLINK 1.9 using the LD-based clumping procedure in the –clump option [29]. The –clump-p1 parameter was used to set the index variant *p*-value threshold.

### Single-nuclei analysis of human DRG

Human DRG snRNA-seq RNA sequencing data was downloaded from NCBI GEO database (GSE168243) in FASTQ format and processed with the cellRanger analysis pipeline v6.1.2 [35]. Nuclei greater than 25% mitochondrial UMIs were discarded. After the filtering step, the gene by cell matrix of raw UMI counts was log-normalized using ‘NormalizeData()’ in SeuratV3 [36]. Samples were integrated using ‘FindIntegrationAnchors()’ and ‘IntegrateData()’ functions in SeuratV3. Then, we scaled the integrated data, performed dimensionality reduction by PCA, and calculated UMAP coordinates and Louvain clustering for all nuclei using SeuratV3. DRG neuron and non-neuronal clusters were identified based on the expression of known cell-type specific markers (SNAP25, UCHL1, RBFOX3, APOE, SPARC, PLP1, PMP22, MPZ1, MBP, PECAM1, VWF, PNPLA2, ADIPOQ). Then, we subsetted DRG neurons to perform analysis to obtain high-resolution clusters within the DRG neuron group. We first removed all non-neuronal nuclei barcodes from the initial clustering and then nuclei that expressed any satellite glial specific transcripts (PLP1 < 1 and MPZ < 1) were removed. The resulting digital gene-expression matrix (DGE) was carried forward for clustering.

Based on previous literature [35, 37–41], we annotated different subsets of large diameter myelinated A-LTMRs using NEFH, PVALB, VSNL1, SLC17A7, CALB1, NTRK3, SCN5A, NTRK2, NECAB2, CNTNAP2, and FAM19A1. Non-peptidergic C-fiber nociceptors (NPs) subsets were annotated using GFRA1, GFRA2, TRPC3, LPAR3, CHRNA3, SST, IL31RA, NPPB, TRPV1, TRPA1, RET, SCN10A, SCN11A, P2RX3, and PLXNC1. C-fiber peptidergic nociceptors (PEPs) subsets were annotated using TAC1, ADCYAP1, GAL, KIT, CALCA, NTRK1, TRPA1, FAM19A1, SCN10a, and SCN11A. Cold thermoreceptors subsets were annotated using TRPM8, TAC1, FOXP2, CDH8, and PENK. We generated pseudo-bulk expression profiles by summing all raw transcript counts per cell type from all five donors to generate a gene by cell type count matrix. We performed CPM (counts per million) normalization on each pseudo-bulk expression profile (cell type by count matrix) using edgeR::cpm() to account for the total number of reads from each cell type.

## Results

### Risk of PN is elevated in taxane-treated patients

We combined time-to-first PN event data from 14 randomized controlled trials spanning a portion of the development path of the PD-L1 inhibitor atezolizumab. The trials were originally intended to evaluate the efficacy and safety of treatment combinations with atezolizumab but also included a substantial number of patients that received chemotherapies as part of their treatment regimen, either in the standard of care arm or in combination with atezolizumab (Additional file 1: Table S1). The trials used similar protocols, and because chemotherapies were used, PN was monitored to find any evidence of elevation of these events when atezolizumab was used in combination. The clinical trials spanned several cancers including renal cell carcinoma (IMmotion151 [11]), triple-negative breast cancer (IMpassion130 [12]), non-small cell lung cancer (IMpower110 [13]/130 [14]/131 [15]/132 [16]/150 [17]), small cell lung cancer (IMpower133 [18]), bladder cancer (IMvigor010 [19]/130 [20]/211 [21]), ovarian cancer (IMagyn050 [22]), melanoma (IMspire170 [23]), and a phase 1 trial in several solid cancers [24]. The patient data could be divided into 30 different clinical trial arms where each arm represented a group of patients receiving a specific treatment at a cancer stage and line of therapy (Additional file 1: Table S1). In total, our study sampled a broad range of contexts where PN events can arise.

Exactly 4900 patients from these 30 different clinical trial arms provided informed consent for genetic data collection, had 30× whole genome germline sequencing data collected, were of European ancestry (EUR > 0.7), and met strict population and genotype data quality control filters (see the “Methods” section). The majority of patients in this cohort 82.2% (4029/4900) were chemotherapy naive (Table 1). PN events in clinical trials were identified as per the clinical trial protocols and graded based on investigator assessed Common Terminology Criteria for Adverse Events (CTCAE) (see the “Methods” section). In all trial arms, at least one patient with genetic data experienced a PN event, and across the clinical trials, a total of 1117 patients with genetic data that experienced PN events were identified. Chemotherapy regimens generally followed a 21-day dosing schedule with few exceptions (Additional file 1: Tables S2 and S3). Eight hundred sixty-eight of the PN events occurred before the 6th cycle of treatment (day 126). The first event the patients experienced was of CTCAE grade 1 (*N* = 709) or grade 2 or more (*N* = 408) respectively (Table 1).

**Table 1:** Cohort characteristics. Per trial arm characteristics are provided in Additional file 1: Table S1

Characteristics	Entire cohort	Taxane-treated	Non-taxane
	*N* = 4900	*N* = 2535	*N* = 2365
**Age**			
> 65	2150	1012	1138
≤ 65	2750	1523	1227
**Sex**			
Male	2746	1068	1678
Female	2154	1467	687
**Prior chemotherapy**			
Naive	4240	2368	1872
Excluding taxanes	167	167	0
Any	493	0	493
**BSA m**^**2**^** median [quartiles]**	1.84 [1.69–1.98]	1.81[1.67–1.96]	1.88 [1.73–2.03]
**Prev. diabetes diagnosis**	743	362	381
**Chemotherapy received**			
Taxane and platinum	2035	2035	0
Platinum only	626	0	626
Taxane only	500	500	0
None	1739	0	1739
**PN events**	1117	1002	115
Grade 1	709	634	75
Grade ≥ 2	408	368	40

Broadly, the cohort could be subdivided into patients that received taxanes (*N* = 2535) as part of their treatment and those that did not receive a taxane (*N* = 2365) as part of the therapeutic regimen in the corresponding clinical trial (Table 1, Additional file 1: Tables S1, S2 and S3). Notably, all patients that received taxanes as part of their chemotherapy treatment regimen were taxane naive. We found that 89.7% (1002/1117) of all first PN events occurred in trial arms where a taxane was used reflecting significantly elevated risk (*p* = 10^−122^, HR = 10.17, 95% CI = 8.38‒12.34) of PN when these agents are used as compared to patients that did not receive taxanes as part of their treatment. Among taxane-treated patients, we found that nab-paclitaxel had a lower risk of PN (*p* = 10^−21^, HR = 0.50, 95% CI = 0.43‒0.58) relative to solvent based paclitaxel. Notably, patients that received a platinum chemotherapy (carboplatin or cisplatin in the absence of a taxane therapy) had only slightly elevated risk of PN relative to patients receiving sunitinib and immunotherapies (*p* = 0.013, HR = 1.60, 95% CI = 1.10–2.32). We additionally examined trial arms where atezolizumab was added to a chemotherapy regimen containing a taxane (IMagyn050, IMpower130/131/150, IMpassion130, see Additional file 1: Table S1). We did not find evidence of elevated risk of PN with the use of atezolizumab in combination with a taxane (*p* = 0.37, HR = 1.06, 95% CI 0.93–1.23). We note that this analysis only examined patients of European ancestry from these trials that consented for genetic data collection, and PN is noted as an adverse drug reaction for atezolizumab when used with the combinations studied in these trials.

We conducted all subsequent analyses using a Cox model stratified by trial arm, an approach also known as one-stage meta-analysis using individual participant data (see the “Methods” section). This approach accounts for the differing baseline hazard for PN in each trial arm. As the likelihood is well specified, this approach also accounts for both rare events and small trial arms [32]. We found that PN events were not associated with sex (*p* = 0.85, HR = 0.98, 95% CI 0.83–1.16) and only modestly associated with patient age (*p* = 0.0004, HR = 1.01, 95% CI 1.005–1.017 per year of age). Grade 1 PN events differ from grade 2 or higher events on the basis of their impact on patient activities of daily living (ADL). We found an association with higher PN grade and age (*p* = 1.06 × 10^−6^, OR = 1.037, 95% CI 1.02–1.05 per year of age, adjusted for sex). We additionally found that risk of PN was associated with body surface area (BSA) (*p* = 7.31 × 10^−8^, HR = 2.13 per m^2^, 95% CI 1.61–2.81) which is used to dose chemotherapies and is consistent with PN’s status as a dose-limiting toxicity. We also assessed whether patients diagnosed with diabetes prior to their enrollment in the trials had higher risk PN. After controlling for age and BSA, we found no evidence of increased risk of PN in diabetic patients of European ancestry from these trials that consented for genetic data collection (*p* = 0.09, HR = 1.15, 95% CI 0.97–1.36).

### Low-frequency variants near *GRID2* are associated with PN risk

**Fig. 1:**
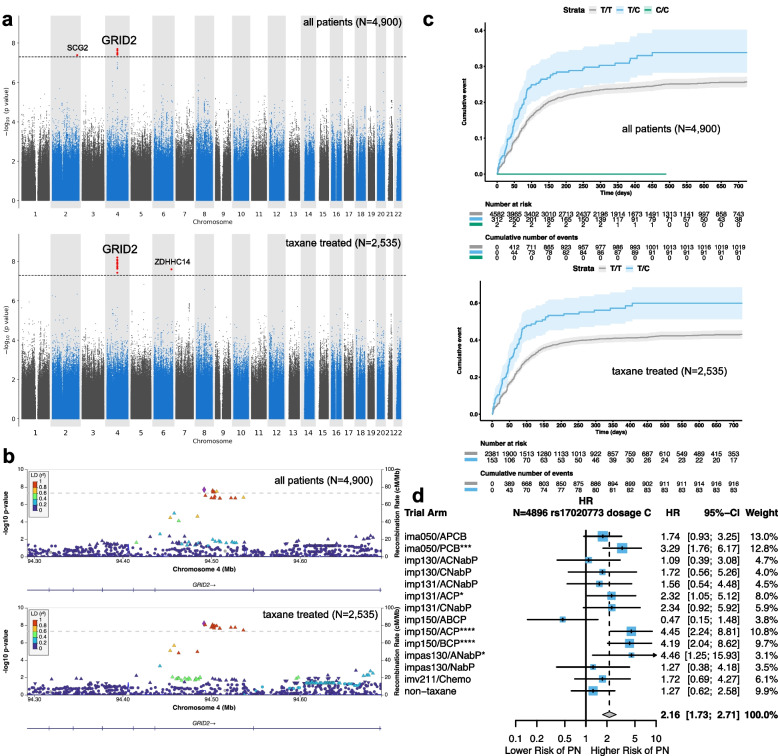
GWAS of time-to-first peripheral neuropathy (PN) event across clinical trials in whole genome sequencing data from European ancestry cancer patients identifies genome-wide significant loci. **a** Manhattan plot showing -log_10_ *p*-values testing for association between common (MAF > 0.01) genetic variants and time-to-first PN event using a Cox proportional hazards model for all patients (top) and for taxane-treated patients (bottom). SNPs reaching genome-wide significance are highlighted in red. Horizontal dashed line shows the genome-wide significance cutoff (*p* < 5 × 10^−8^). The GRID2 locus is indicated above. **b** Locus zoom plot around the GRID2 intron locus for all patients (top) and for taxane-treated patients (bottom). The colors indicate the strength of linkage disequilibrium (*r*.^2^) relative to the index SNP (rs17020773) shown as a purple diamond. **c** Cumulative incidence plot showing the association between dosage of the effect allele of rs17020773 and time-to-PN event for all patients (top) and for taxane-treated patients (bottom). Shaded regions designate the 95% confidence intervals around the cumulative incidence curves. Note that the 2 homozygous carriers of the rs17020773 variant were from trial arms that did not receive chemotherapies. **d** Forest plot illustrating the 95% confidence intervals around the hazard ratio (HR) associating the dosage of the alternate C allele of rs17020773 to time to first PN event. The non-taxane trial arms have been grouped. Trial arm and treatment abbreviations are provided in Table 1. The inverse variance weighted HR is provided as a diamond. *p < 0.05, **p < 0.01, ***p < 0.001, ****p < 0.0001

We conducted a GWAS for time-to-first PN event using 8,411,915 variants with minor allele frequency (MAF) ≥ 0.01 (Fig. 1a). Because we used WGS data, the variants in the low-frequency range MAF < 0.05 and MAF ≥ 0.01 were directly genotyped. In contrast, with array based genotyping most of the variants in this range would be imputed with varying degrees of accuracy. Given that the majority of all of the first PN events 89.7% (1002/1117) occurred in trial arms where a taxane was used, we also conducted a GWAS using only patients that received a taxane as part of their treatment regimen (Fig. 1a). We found that the test statistics of this GWAS of the entire cohort (genomic inflation factor, *λ*_gc_ = 1.004) and for the taxane-treated subcohort (*λ*_gc_ = 1.005) were well calibrated confirming that the stratified Cox model accounted for the differing baseline hazard in each trial arm, rare PN events, small trial arms, low-frequency variants, and any population stratification (Fig. S1, Additional file 1: Tables S1, S2 and S3).

We identified one locus reaching genome-wide significance (*p* < 5 × 10^−8^) in all patients (link: https://my.locuszoom.org/gwas/74850) and within the taxane-treated subcohort (Fig. 1a, link: https://my.locuszoom.org/gwas/743668, Additional file 1: Table S4). The locus was located on chromosome 4 in intron 13 of *GRID2* (Fig. 1b). The index SNP for the locus was rs17020773 (T/C, with C being the allele associated with higher PN risk, *p* = 2.03 × 10^−8^, HR = 1.85, 95% CI = 1.50–2.31, risk allele frequency = 0.032, HGVS: NC_000004.12:g.93570776 T > C GRCh38.p13 chr 4). We found that the association at rs17020773 was stronger within the taxane-treated subcohort (*p* = 6.36 × 10^−9^, HR = 1.96, 95% CI = 1.56–2.47). We confirmed that the coefficient corresponding to genotype status in the Cox model did not violate the proportional hazards assumption for rs17020773 within the entire cohort (zph test *p* = 0.55) and in the taxane-treated subcohort (zph test *p* = 0.64). We found that the effect of the locus was large enough that it was evident in a cumulative incidence plot across all the patients and within the taxane-treated subcohort (Fig. 1c). We confirmed that both associations remained genome-wide significant after adjusting for BSA for all patients (*p* = 6.92 × 10^−9^, HR = 1.91, 95% CI = 1.53–2.38) and in the taxane-treated subcohort (*p* = 2.28 × 10^−9^, HR = 2.00, 95% CI = 1.60–2.52) indicating that dosing could not account for the associations we observed. We also confirmed that the association with rs17020773 was evident when we considered only grade 1 events (*p* = 4.39 × 10^−6^, HR = 1.82, 95% CI 1.36–2.42) or grade 2 or higher events (*p* = 1.41 × 10^−6^, HR = 2.25, 95% CI = 1.61–3.13). Last, we stratified the data set and considered the hazard ratio within each trial arm separately to demonstrate that no single trial arm was driving the association at rs17020773 we observed (Fig. 1d).

We examined whether any gene regulatory evidence supported that this locus might operate through *GRID2*. Single cell multiomics can link chromatin accessibility to gene expression through the analysis of peak-gene co-variation across cells. We identified three open chromatin peaks within the rs17020773 locus in a recent single-cell multi-omic atlas [42]. Only two protein coding genes, *GRID2* and *ATOH1*, had canonical transcription start sites (TSSs) within 土500kb of the locus. We found that the mRNA expression of *GRID2* to be positively correlated with all three chromatin accessibility peaks whereas *ATOH1* was positively correlated with only one (Additional file 1: Fig. S2). These data provide evidence that supports *GRID2* as the gene mediating PN risk through the locus indexed by rs17020773.

We additionally observed a variant 126 kb downstream of the transcription start site of *SCG2* associated with risk of PN at genome-wide significance in the entire cohort: rs115575220 (G/T with T being the risk allele, *p* = 4.15 × 10^−8^, HR = 2.44, 95% CI = 1.77–3.35, risk allele frequency = 0.012). However, rs115575220 was not genome-wide significant within the taxane-treated subcohort. Within the taxane-treated subcohort, rs191482247, a variant within the first intron of *ZDHHC14*, was associated with risk of PN at genome-wide significance (A/G with G being the risk allele, *p* = 2.54 × 10^−8^, HR = 2.29, 95% CI = 1.71–3.06, risk allele frequency = 0.015), but not within the entire cohort. At each of these two loci, *SCG2* and *ZDHHC14* only a single variant of lower frequency (close to our cutoff of 0.01) passed genome-wide significance. There were no variants in strong linkage disequilibrium (LD) (> 0.8) and only a limited number of variants that were in moderate (> 0.4) LD with these variants (Additional file 1: Fig. S3). A larger, more homogeneous, cohort may be required to confirm these associations.

Prior array-based genotyping studies have identified common variants associated with CIPN [5–10]. We asked whether these prior associations replicated in our study. We tested 18 variants previously reported as associated with CIPN either in taxane or platinum-based chemotherapy patients reported in a recent review in addition to a recently reported variant near *S1PR1* [9, 10]. We found that only the variants with suggested candidate genes GPR177 (rs3125923) and *FZD3* (rs7001034) replicated in our cohort at a nominal *p*-value of *p* < 0.05 (Additional file 1: Table S5). *GPR177* is expressed in dorsal root ganglia and has recently been shown to activate *TRPV1* ion channels in a murine model of diabetic neuropathic pain [43]. Substantial genetic evidence supports *FZD3* as a causal gene for Charcot-Marie-Tooth hereditary neuropathy [44].

Heredity neuropathy may also occur in individuals over their lifetime. The CIPN events we observed may arise out of an underlying genetic risk for these lifetime neuropathy events. We constructed a polygenic risk score (PRS) model using PRS-CS [34], a Bayesian beta-shrinkage method, that used summary statistics from a recent GWAS of hereditary neuropathy derived from individuals in the UK Biobank (GWAS catalog GCST90038643) [33]. We used this PRS model to score cancer patients within our cohort and asked whether this score was associated with risk of PN events during cancer treatment. We found no association between a PRS of hereditary neuropathy and PN events within cancer patients (*p* = 0.99, HR = 1.00, 95% CI = 0.94–1.06 per unit normalized PRS). This analysis suggests that the genetic etiology of these events is distinct. Consistent with this observation, we found that rs17020773 alone was not associated with hereditary neuropathy within this GWAS (*p* = 0.46). A PRS derived from our GWAS for PN events can also be used as a predictor in independent cohorts of European ancestry cancer patients. To enable this, we provide a PRS model derived from our cancer patient cohort as supplementary data using PRS-CS (Additional file 2: Table S6) [34]. We additionally provide a simplified PRS derived from our GWAS using LD-pruning with a cutoff of *p* < 10^−4^ (Additional file 3: Table S7) and *p* < 10^−6^ for index variants (Additional file 4: Table S7).

### Rare coding variant burden identifies *GPR68* as a PN susceptibility gene

Given the availability of whole genome sequencing data in our cohort, we could interrogate rare coding variants for their association with time-to-PN. To do so, we aggregated protein altering variants with MAF < 0.01 in the gene into a burden score and tested for its association with time-to-PN using a Cox model at a gene-level (see the “Methods” section). Similar to the common variant analysis above, we performed the genome-wide burden test scans using (1) all patients and (2) taxane-treated subcohort. For the all patients’ analysis, we limited such survival burden tests to 13,263 genes where there were at least 30 carriers. As the Cox model was stratified by trial arm, we confirmed that the burden test statistics were well calibrated for all patients’ analysis (*λ*_gc_ = 1.009, Additional file 1: Fig. S4). Burden test statistics were also well calibrated for 9738 genes with at least 30 carriers tested within the taxane-treated subcohort (*λ*_gc_ = 1.017, Additional file 1: Fig. S4).

We identified one gene, *GPR68*, a pH sensitive G-protein coupled receptor (GPCR) that reached the exome-wide significance cutoff (*p* < 2.5 × 10^−6^) in both analyses. In all patients analysis, *GPR68* had 51 rare variant carriers, and rare variant burden that was significantly associated with time-to-PN (*p* = 1.59 × 10^−6^, HR = 2.44, 95% CI = 1.52–2.72). We found that the association remained after adjusting for BSA (*p* = 6.58 × 10^−7^, HR = 2.09, 95% CI = 1.56–2.79) indicating that dosing could not account for the association we observed. We additionally confirmed that the variable corresponding to the rare variant burden in the Cox model did not violate the proportional hazards assumption (zph test *p* = 0.21, after BSA adjustment *p* = 0.24). In the subset of patients that were treated with taxanes, there were 33 *GPR68* rare variant carriers, and the effect of these rare variants on time-to-PN could be seen before (*p* = 3.47 × 10^−8^, HR = 2.25, 95% CI = 1.69–3.01, zph test *p* = 0.88) and after adjusting for BSA (*p* = 1.08 × 10^−8^, HR = 2.29, 95% CI = 1.71–3.06, zph test *p* = 0.88). We also confirmed that the burden of rare coding variants in *GPR68* were associated with grade 1 events alone (*p* = 0.006, HR = 2.06, 95% CI = 1.36–3.13) and with grade 2 or higher events (*p* = 1.12 × 10^−6^, HR = 2.80, 95% CI = 1.84–4.22).

**Fig. 2:**
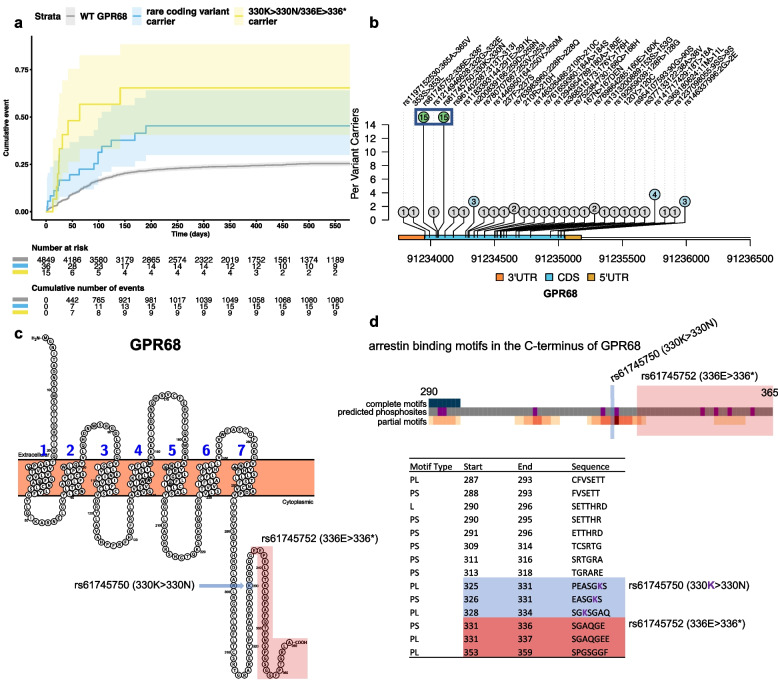
Rare coding variation in *GPR68* is associated with risk of chemotherapy-induced peripheral neuropathy. **a** Cumulative incidence plot showing the association between the burden of rare coding variants in *GPR68* and time-to-PN event in all trial arms. Shaded regions designate the 95% confidence interval around the cumulative incidence curves. **b** Lollipop plot of rare coding variants in *GPR68* that were used for burden testing with time-to-PN event in the entire clinical trial cohort. The *y*-axis of the plot provides the number of patients that carried the rare coding variant as designated by rsID and coding sequence consequence along the top of the plot. rs61745750 (330 K > 330N) and truncating variant rs61745752 (336E > 336*) is highlighted by the blue square. **c** Protein plot illustrating the trans-membrane domains of *GPR68* as well as the position of rs61745750 (330 K > 330N) highlighted in blue and rs61745752 (336E > 336*) highlighted in red on the C-terminus of the protein. **d** Gray squares illustrate amino acids starting at position 290 in *GPR68* interspersed with purple squares that designate predicted phosphorylation sites. Complete arrestin binding motif matches shown in the first row of blue squares. Partial motif matches are shown in peach color below where darker squares correspond to amino acids involved in more binding motifs. The position of rs61745750 (330 K > 330N) is highlighted in blue and rs61745752 (336E > 336*) truncation is highlighted in red. Positions and sequence of the motif matches are provided in the table where PL = partial long, PS = partial short, L = complete long. The lysine substituted by asparagine is shown across motif matches in purple. Motifs removed by rs61745752 (336E > 336*) are highlighted in red

We next examined the cumulative incidence plot showing the association between burden of rare variants in *GPR68* and time-to-PN in all patients (Fig. 2a) and in taxane-treated patients (Additional file 1: Fig. S5). We observed that 15 out of 51 patients carrying any rare coding variant in this gene in all treatment arms and 11 out of 33 patients carrying any rare coding variant in the taxane arms had two variants in the C-terminus of *GPR68*, rs61745750 (330 K > 330N, HGVS:ENSP00000498702.1:p.Lys330Asn, GRCh38.p13) and truncating variant rs61745752 (336E > 336*, HGVS:ENSP00000498702.1:p.Glu336Ter, GRCh38.p13), that were strongly associated with risk of PN and in perfect linkage disequilibrium (LD) (Fig. 2b, c). We confirmed that these variants were also in perfect LD within the Haplotype Reference Consortium imputation panel (frequency of 0.00107) [45]. Within gnoMAD v2.1.1, these variants were at even lower allele frequency in non-European ancestry individuals, only 7 non-European carriers as opposed 123 European ancestry carriers.

Typical genome-wide burden tests, such as the one we performed, assume independence between rare variants while aggregating them into a burden score. We therefore also computed burden test statistic counting rs61745750 and rs61745752 as a single variant for all patients (*p* = 1.44 × 10^−5^, HR = 2.49, 95% CI = 1.65–3.76) and for taxane-treated patients (*p* = 3.10 × 10^−6^, HR = 2.70, 95% CI = 1.78–4.10). Using this modified burden score, *GPR68* was still strongly associated with time-to-PN. We also found that carriers of the rs61745750 and rs61745752 were 4.48 times more likely to develop PN as compared to patients that had no rare coding variants in *GPR68* (HR = 4.48, 95% CI 2.25–8.91). In taxane-treated patients, the difference in risk between these patients was higher (HR = 5.62, 95% CI 2.87–11.00). We next excluded rs61745750 (330 K > 330N) and rs61745752 (336E > 336*) and found that the remaining *GPR68* rare variants were nominally associated with time-to-PN across all patients (*p* = 0.015, HR = 1.89, 95% CI = 1.12–3.16) and within taxane-treated patients (*p* = 0.009, HR = 2.02, 95% CI = 1.19–3.45) but at approximately half of the hazard ratio as carriers of rs61745750 and rs61745752. Taken together, the hazard ratios illustrate an additive relationship between the number of rare variants in *GPR68* and risk of PN—supporting the inclusion of rs61745750 and rs61745752 as two variants in the burden score.

The C-terminus of a GPCR is important for its function [46]. GPCR ligands control phosphorylation patterns at the C-terminus by modulating interactions of a GPCR with various G protein receptor kinases, which phosphorylate different sets of GPCR residues. Depending on these phosphorylation patterns, the GPCR can recruit arrestin to block GPCR signaling through internalization of the receptor. *Px(x)PxxP* amino acid motifs in the C-termini of GPCRs are thought to encode phosphorylation sites that are required for arrestin binding [47] (*P* in the motif corresponds to serine or threonine and *x* any amino acid and the parentheses denote an optional match.) Using PhosCoFinder [47], we identified three partial motif matches that were removed by the truncating rs61745752 (336E > 336*) variant, three additional partial motif matches that were altered by the rs61745750 (330 K > 330N) substitution, and loss of several predicted phosphorylation sites in the C-terminus of *GPR68* (Fig. 2d). The disrupted motif matches were non-overlapping between the two variants. This indicates that, while they are on the same haplotype, the variants may have distinct effects on *GPR68*.

### *GPR68* is expressed in PEP1 sensory neurons in human DRG

The symptomatic profile of CIPN implicates the peripheral nervous system and sensory neurons in human dorsal root ganglia (DRG) [48]. DRG are composed of multiple neuronal subtypes that are tuned to respond to different stimuli and have unique chemical and electrophysiological properties. Important for pain are the C-nociceptors, unmyelinated neurons that sense unpleasant sensations such as extreme heat or cold. We re-analyzed data from a publication that collected single-nucleus RNA-seq (snRNA-seq) data from human DRG from *n* = 5 donors (see the “Methods” section) [35]. Using this data, we were able to label all major sensory neuron cell types as well as a small number of non-neuronal cell types.

**Fig. 3:**
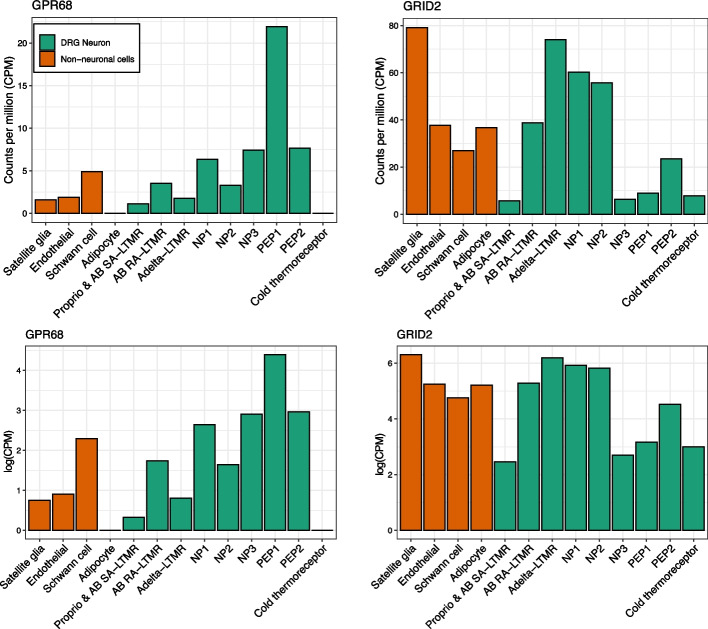
Expression patterns of *GPR68* and *GRID2* across cell types in human dorsal root ganglia (DRG) [35]. The *y*-axis represents counts per million (CPM) (top) and log(CPM) (bottom) of the pseudo-bulk expression of genes in the annotated cell types in the human DRG from *n* = 5 donors. DRG neurons are designated in green whereas non-neuronal cells are shown in orange. DRG neurons are molecularly classified into different groups: heavily myelinated limb proprioceptors and A-fiber low-threshold mechanoreceptors (LTMRs) that process innocuous touch sensation and proprioception; C-fiber non-peptidergic (NP) nociceptors that process nociception and pruritus; non-myelinated C-fiber and lightly myelinated A-fiber peptidergic (PEP) nociceptors which process inflammatory heat-induced and sharp pinprick nociception; cold thermoreceptors which are A-fiber and C-fiber nociceptors that process cold sensation

Within these data, we examined the relative expression levels of *GPR68* across cell types (Fig. 3). Consistent with previous reports by antisense probe labeling and Western blot that *GPR68* is expressed in nociceptor neurons in DRG, we found that *GPR68* had the highest expression in PEP1 sensory neurons relative to all other labeled cell types [49]. We additionally confirmed this pattern was conserved in mouse and macaque (Additional file 1: Fig. S6). PEP1 neurons are a subtype of C-nociceptors that express the mustard-responsive channel TRPA1 as well as high levels of genes that encode the pain-eliciting peptides substance P (*TAC1*) and CGRP (*CALCA*). We also examined the expression pattern of *GRID2* in human DRG. In contrast to *GPR68*, *GRID2* had higher expression in DRG overall and noticeably higher expression in satellite glia (Fig. 3). This pattern was conserved in mice in a data set that compared expression in satellite glial cells to overall bulk expression in DRG (Additional file 1: Fig. S7) [50]. Satellite glial cells wrap the soma of sensory neurons and are important for neuronal homeostasis and response to neuronal stress [51].

## Discussion

Our study reveals a potential link between *GPR68* and CIPN. We identified two rare coding variants in the C-terminus of this pH sensing GPCR associated with risk of CIPN in patients receiving chemotherapies as part of their treatment regimens. We were able to detect these variants due to their large effect size in taxane-treated patients (> 5× increased risk relative to patients without any *GPR68* coding alleles). Using single cell RNA-seq data from human DRG, we found that the highest relative expression of *GPR68* within DRG was in PEP1 sensory neurons, a pattern that was conserved across species. These neurons are nociceptors that respond to intense mechanical or thermal stimuli. Sensitivity of these neurons to chemotherapy may underlie the paresthesia and burning sensations experienced during CIPN.

The CIPN associated rare coding variants we found led to loss and alteration of predicted phosphorylation sites and arrestin binding motifs in the C-terminus of *GPR68*. Loss of these sites and disruption of these motifs can be expected to block arrestin mediated receptor internalization, prolonging activation of *GPR68* signaling to sensitize PEP1 sensory neurons to chemotherapies. Thus, the variants are predicted to be gain of function. Consistent with this model, a recent study has provided evidence that the C-terminal truncating variant rs61745752 (336E > 336*) alone prevents receptor internalization in acidic conditions in transfected HEK293T cells [52]. GPCRs are highly druggable, and several inhibitors and allosteric ligands of *GPR68* are known [52, 53]. In contrast to our findings which suggest inhibition of *GPR68* signaling might be beneficial for CIPN, *GPR68* overexpression was found to be neuroprotective in a model of sevoflurane-induced neurotoxicity [54]. Further studies are needed to resolve the role *GPR68* plays in neuroprotection and pain.

We identified a locus in intron 13 of *GRID2* associated with CIPN risk both in the entire cohort and in taxane-treated patients. We found gene regulatory evidence that supports *GRID2* as the candidate gene. However, given context dependent activity of many genetic variants, further studies are needed to link this locus to *GRID2* as the locus may affect genes through distal interactions. *GRID2* is a member of the glutamate receptor family. Although glutamate plays an important role in pain perception, *GRID2* is not known to bind glutamate and is considered an orphan receptor [55]. Within DRG, we found that *GRID2* was expressed in satellite glial cells as well as sensory neurons. Maintenance of neuronal homeostasis and response to neuronal stress requires bidirectional communication between glial cells and neurons through ion channels and receptors [51]. *GRID2* regulation might mediate such interactions during chemotherapy treatment. Consistent with this hypothesis, satellite glial cells have been implicated in CIPN models in mice [56].

Our proposed models for the impact of genetic variants in *GPR68* and near *GRID2* rely on the expression patterns of these genes in human DRG. When contrasted with other human tissues, *GPR68* has higher expression in the small intestine, and lung and *GRID2* has higher expression in non-neuronal tissues [57]. This is consistent with observations in rodent studies [58]. Notably, these genes have not been observed as differentially expressed in pain experiments [59]. Expression quantitative trait loci (eQTL) studies in human DRG have been limited to higher frequency variants than assayed by whole genome sequencing, and thus, no eQTL evidence exists for these variants [60]. While the symptomatic profile of CIPN suggests that the DRG expression patterns of these genes are important, further studies are needed to link these variants to the function of these genes in the chemotherapy-induced pathophysiology of DRG.

The PN association signal we found in intron 13 of *GRID2* replicates across clinical trial arms and cancers. Replication of the rare coding variant burden signal in *GPR68* will be more challenging as it will require construction of another large whole genome sequencing cohort of patients that have detailed follow-up information and time of diagnosis of PN after the start of treatment. This may prove challenging outside of the context of clinical trials. Our study also focused on patients of European ancestry because they represented the majority of patients in the clinical trials where WGS data was available and GWAS within ancestries are less susceptible to population stratification. Studies of CIPN in non-European ancestries will be enabled by ongoing efforts to increase the diversity of patients enrolled in clinical trials and may provide insight into population specific variants associated with CIPN.

In each of the clinical trials we examined, PN events were identified from the safety data reported according to study protocols. PN events are identified either following reported symptoms or observed clinical or diagnostic findings by the study investigator who then grades the event on the basis of the NCI CTCAE. Under the NCI CTCAE, one of the primary factors distinguishing grade 1 PN events from grade 2 or higher events is the impact on a patient's activities of daily living (ADL). Grade 1 events are thought to be more challenging to detect, and there is a potential for under-reporting of grade 1 PN events. In general, the consensus in the field supports the use of patient-reported outcomes (PROs) for outcome measures in clinical trials evaluating therapies for treatment of CIPN [61]. However, whether PROs provide advantages in biomarker studies of CIPN, such as the one we conducted here, remains a subject of debate [62]. This debate is ultimately linked to the subjectivity of assessing PN by study investigators and by patients—which can differ across cohorts and trials.

This subjectivity of assessing PN may introduce bias, which we addressed by aggregating and meta-analyzing data from several clinical trials from several treatment contexts where similar study protocols were used. Nevertheless, substantial care must still be used in comparing associations in any one study to another as differences in how PN events are detected need to be considered. We also note that grade 1 PN events are not dose-limiting because they have no impact on ADL. Yet, our study considered all events to increase power to detect genetic associations. We find evidence that both the genetic variants near *GRID2* and in *GPR68* increase risk of both grade 1 and grade 2 or higher events. Yet, larger cohorts are needed to determine whether genetic risk factors have distinct effects on PN events of differing grades.

Platinum and taxane chemotherapies have different mechanisms of action and may contribute to CIPN through differing mechanisms. An implicit assumption of a stratified Cox model that underlies our study is that the average estimated hazard ratio due to a genetic variant is similar across strata—here trial arms. This assumption might not be true for the entire cohort and may also lead to loss of statistical power due to the inclusion of patients receiving these two therapies. The treatment combinations with taxanes within our cohort used differing dosing schedules and regimens. Ideally, a study where treatment dosage is fully controlled would address these limitations. However, constructing such a cohort where patients have consented for genetic data collection could prove to be challenging.

There may also exist common genetic risk factors that render sensory neurons susceptible regardless of therapy. We found that the *GRID2* locus to be associated with PN both within the entire cohort and within the taxane-treated subcohort. Yet, the variants near *SCG2* and *ZDHHC14* only reached genome-wide significance within the entire cohort and subcohort respectively. Whether these variants are common or distinct genetic risk factors for different classes of chemotherapies may only become apparent in larger studies with more homogenous treatment regimens. Our findings suggest that such genetic studies could yield further insights into CIPN.

In summary, our study illustrates the promise of whole genome sequencing in patient populations that receive drugs with known dose-limiting toxicities. Findings from these studies provide an opportunity to elucidate the genetic architecture of toxicities and identify rare genetic variants associated with toxicity risk. Given that drug targets with supporting human genetic evidence are more likely to lead to approved drugs, this approach has the potential to power discovery of drug combinations that provide a more favorable benefit/risk profile for patients [63].

## Conclusions

By conducting a study of the genetic basis of CIPN risk using 30× germline WGS data from 4900 European ancestry cancer patients from 14 clinical trials, the largest such study to date, we report a genome-wide significant locus associated with time-to-onset of PN indexed by the variant rs17020773. The variants in this locus implicate *GRID2* as a candidate gene in CIPN risk. Using rare coding variant burden analysis, we identified *GPR68*, a pH-sensitive GPCR, as a CIPN susceptibility gene. The coding burden signal was mainly driven by two variants, rs61745750 (330 K > 330N) and rs61745752 (336E > 336*) in the C-terminus of *GPR68* that disrupt predicted arrestin binding motifs. These findings provide human genetic evidence that supports further study of the role of both *GRID2* and *GPR68* in CIPN risk and prevention.

## Supplementary Information

**Additional file 1: Figure S1.** QQ-plot of *p*-values from a common variant GWAS of time to first peripheral neuropathy for all patients *N*=4,900 and for the taxane treated subcohort, *N*=2,535. **Figure S2.** Correlation between chromatin accessibility peaks within the rs17020773 locus and the expression of *GRID2* and *ATOH1* in a multiomic atlas. **Figure S3.** Locus zoom plots for rs115575220 in all patients and for rs191482247 in taxane treated patients. **Figure S4.** QQ-plot of p-values from a rare variant burden test of time to first peripheral neuropathy for the entire cohort *N*=4,900 and for taxane treated patients *N*=2,535. **Figure S5.** Cumulative incidence plot for PN events in taxane treated patients stratified by rare coding variant burden in *GPR68*. **Figure S6.** Relative expression patterns of *GPR68* in different DRG cell types in macaque and mice. **Figure S7.** Expression of *GRID2* in bulk RNA-seq from human DRG as compared to sorted satellite glial cells in mice. **Table S1.** Frequency and grade of PN events in cancer patients of European ancestry across 14 randomized controlled trials testing immunotherapy and chemotherapy combinations. **Table S2.** Taxane Regimens. **Table S3.** Platinum Chemotherapy Regimens. **Table S4.** Low frequency variants that were associated with risk of PN at *p* < 5・10-8 across *N*=4,900 cancer patients or within the *N*=2,535 taxane treated cohort. **Table S5.** Evidence for Replication of Variants Previously Associated with CIPN.

**Additional file 2: Table S6.** PRS for CIPN computed using PRS-CS beta-shrinkage. Column descriptions: CHR Chromosome of PRS variant; SNP genetic variant rsID; BP Base pair position on chromosome in the CHR column; EffectAllele Allele associated with PN risk, typically the alternative allele; OtherAllele Allele not associated with risk, typically the reference allele; EffectSize Effect size associated with copies of the risk allele in the EffectAllele Column.

**Additional file 3: Table S7.** Simplified PRS computed using LD-pruning with *p* < 10^−4^. The same column descriptions for Additional file 2: Table S6 apply to this PRS.

**Additional file 4: Table S8.** Simplified PRS computed using LD-pruning with *p* < 10^−6^. The same column descriptions for Additional file 2: Table S6 apply to this PRS.

## Data Availability

Summary statistics for the common variant GWAS for the entire cohort are available for download here: https://my.locuszoom.org/gwas/74850. Summary statistics for the taxane-treated subcohort are available for download here: https://my.locuszoom.org/gwas/743668. Qualified researchers may request access to individual patient data used in this study through Roche’s data sharing platforms in accordance with the Global Policy on Sharing of Clinical Study Information: https://www.roche.com/innovation/process/clinical-trials/data-sharing/. To ensure compliance with legal, data retention, and patient confidentiality obligations in the informed consent forms (ICFs), the whole genome sequencing data collected (in VCF or BAM/FASTQ formats) cannot be hosted on a public, controlled access repository and will be made available to individual requesters on completion of a data sharing agreement with Roche/Genentech. Requests for access to whole genome sequencing data should be made to the corresponding authors by email. The planned research with the requested data will be reviewed by the Roche Pharma Repository Governance Committee to assess its scientific merit and to ensure it is in scope of patient ICFs.

## Abbreviations

PN: Peripheral neuropathy
CIPN: Chemotherapy-induced peripheral neuropathy
GWAS: Genome wide association study
BSA: Body surface area
DRG: Dorsal root ganglia
PRS: Polygenic risk score
WGS: Whole genome sequencing
LD: Linkage disequilibrium
MAF: Minor allele frequency
ADL: Activities of daily living
NCI CTCAE: National Cancer Institute Common Terminology Criteria for Adverse Events
PRO: Patient-reported outcomes
AE: Adverse event
snRNA-seq: Single nucleus RNA-seq
