# Supplementary material for: Whole genome sequencing across clinical trials identifies rare coding variants in GPR68 associated with chemotherapy-induced peripheral neuropathy
Source: Genome Med. 2023 Jun 21;15:45. doi: 10.1186/s13073-023-01193-4 (PMC10286344; doi:10.1186/s13073-023-01193-4)
Supplement: Supplementary file 1 — Additional file 1: Figure S1. QQ-plot of p-values from a common variant GWAS of time to first peripheral neuropathy for all patients N=4,900 and for the taxane treated subcohort, N=2,535. Figure S2. Correlation between chromatin accessibility peaks within the rs17020773 locus and the expression of GRID2 and ATOH1 in a multiomic atlas. Figure S3. Locus zoom plots for rs115575220 in all patients and for rs191482247 in taxane treated patients. Figure S4. QQ-plot of p-values from a rare variant burden test of time to first peripheral neuropathy for the entire cohort N=4,900 and for taxane treated patients N=2,535. Figure S5. Cumulative incidence plot for PN events in taxane treated patients stratified by rare coding variant burden in GPR68. Figure S6. Relative expression patterns of GPR68 in different DRG cell types in macaque and mice. Figure S7. Expression of GRID2 in bulk RNA-seq from human DRG as compared to sorted satellite glial cells in mice. Table S1. Frequency and grade of PN events in cancer patients of European ancestry across 14 randomized controlled trials testing immunotherapy and chemotherapy combinations. Table S2. Taxane Regimens. Table S3. Platinum Chemotherapy Regimens. Table S4. Low frequency variants that were associated with risk of PN at p < 5・10-8 across N=4,900 cancer patients or within the N=2,535 taxane treated cohort. Table S5. Evidence for Replication of Variants Previously Associated with CIPN. [file 13073_2023_1193_MOESM1_ESM.pdf]

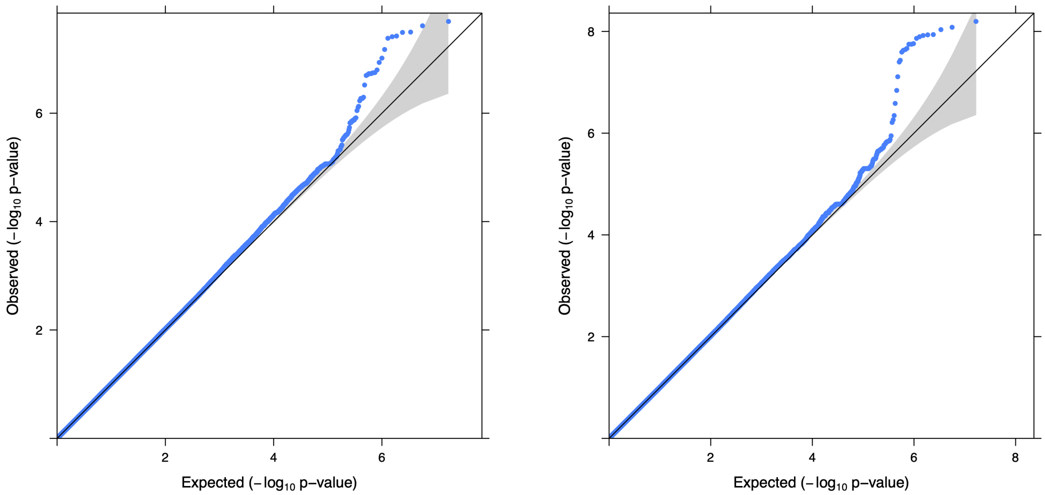


**Figure S1.** QQ-plot of p-values from a common variant GWAS of time to first peripheral neuropathy for all patients N=4,900 (left) and for the taxane treated subcohort, N=2,535.(right). Each point represents a single variant. Genomic inflation factor $\lambda_{gc}$=1.004 (all patients) and $\lambda_{gc}$=1.005 (taxane treated subcohort). Gray region designates the 95% confidence interval around the null distribution of p-values.

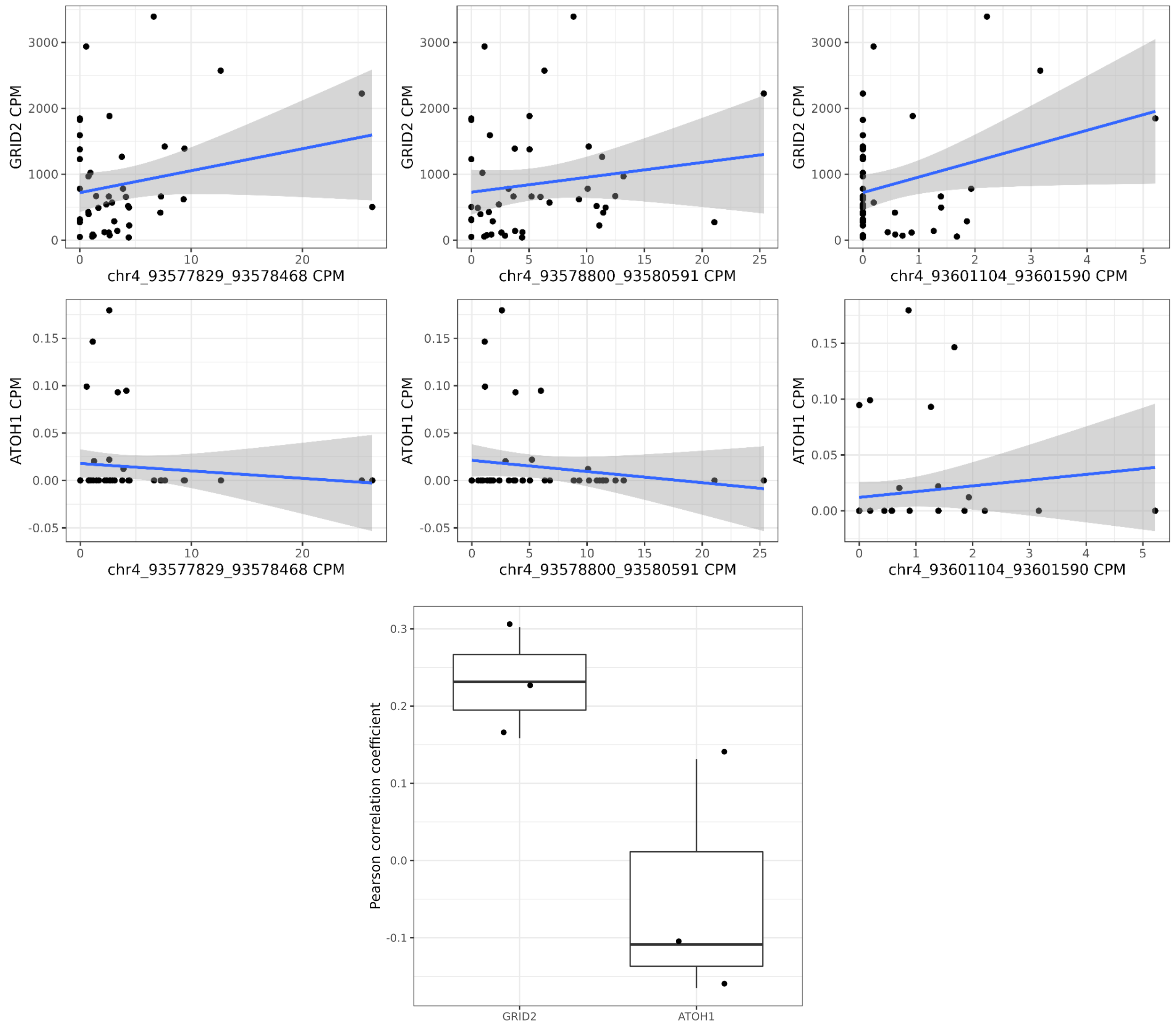


**Figure S2.** Top panel, each point represents a cell type and each subpanel shows the relationship between gene expression and peak accessibility in the rs17020773/*GRID2* intron locus, which contains two protein-coding genes within 1MB (+/- 500KB) and three accessible chromatin peaks [1]. For each cell type classified in the original publication, counts were summed across all genes and all peaks to generate pseudo-bulk profiles for gene expression and chromatin accessibility respectively, then profiles were normalized for read depth by dividing by the total and multiplying by $10^6$ (CPM normalization). Bottom panel, boxplot of each of the correlations shown in the top panels (p = 0.05, two-sided t-test). All 3 regions showed a positive correlation with the expression of *GRID2* whereas only 1 region was positively correlated with *ATOH1*.

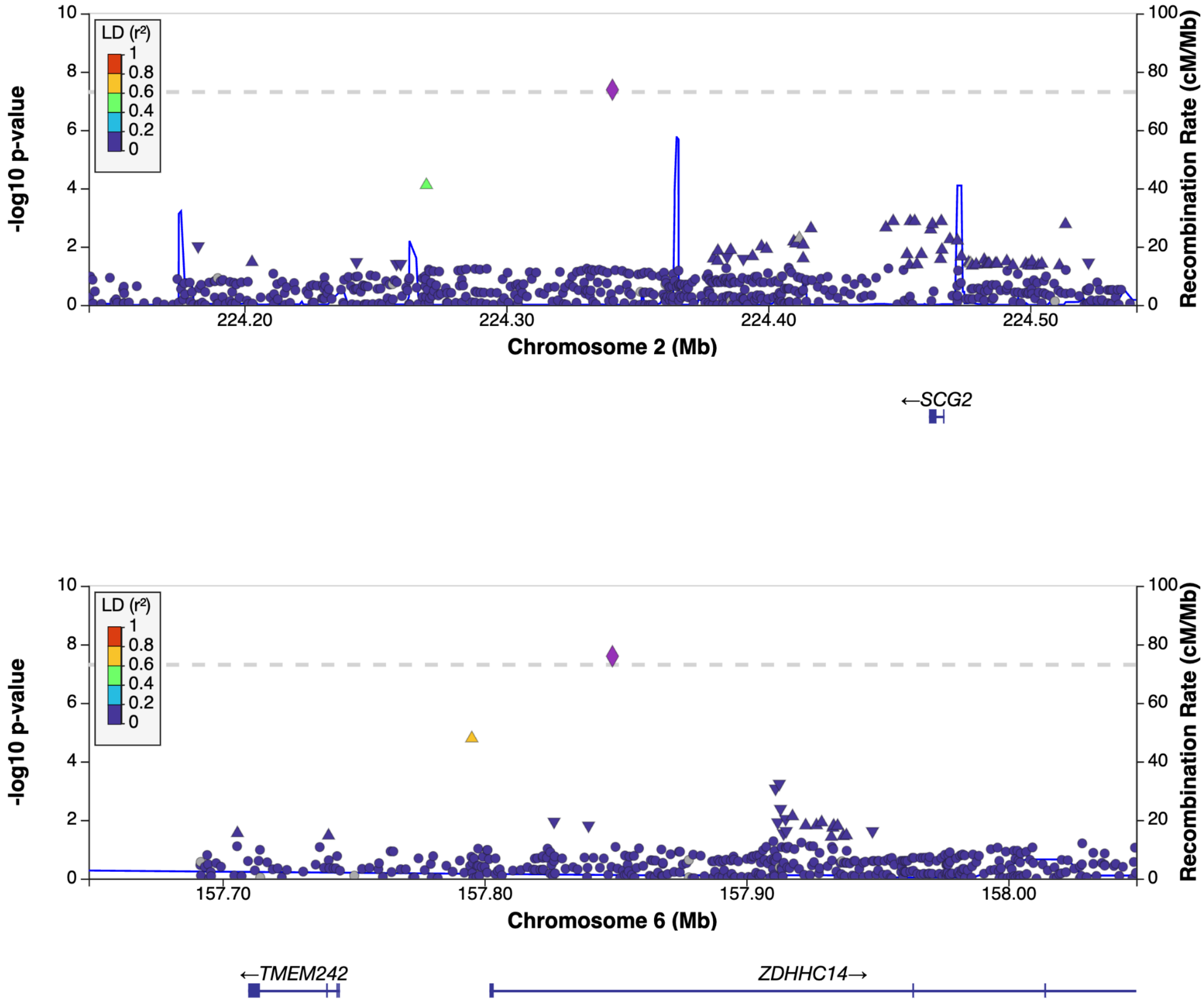


**Figure S3.** Locus zoom plots for rs115575220 in all patients (top) and for rs191482247 in taxane treated patients (bottom). The colors indicate the strength of linkage disequilibrium ($r^2$) relative to the index SNP shown as a purple diamond.

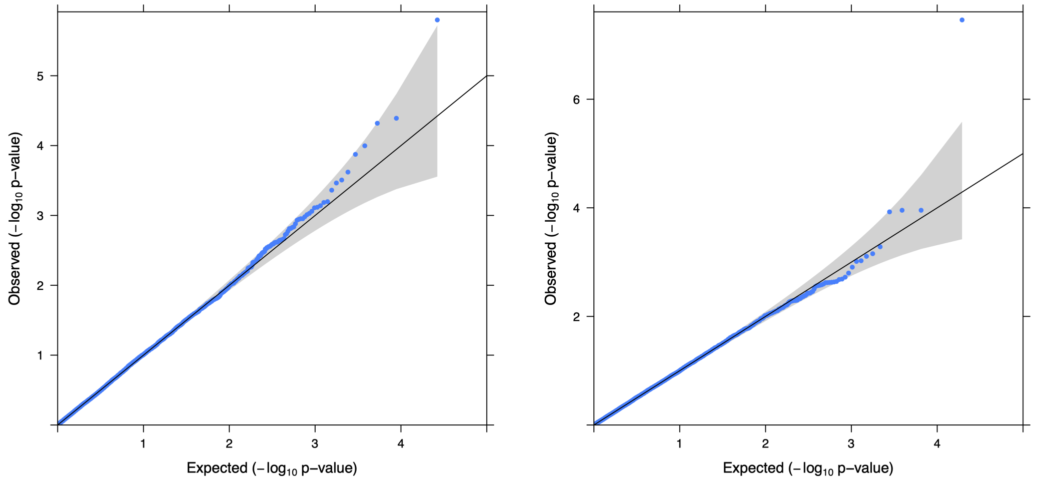


**Figure S4.** QQ-plot of p-values from a rare variant burden test of time to first peripheral neuropathy for the entire cohort N=4,900 (left) and for taxane treated patients N=2,535 (right). Each point represents a gene. $\lambda_{gc}$=1.009 (left) and $\lambda_{gc}$=1.017 (right). Gray region designates the 95% confidence interval around the null distribution of p-values.

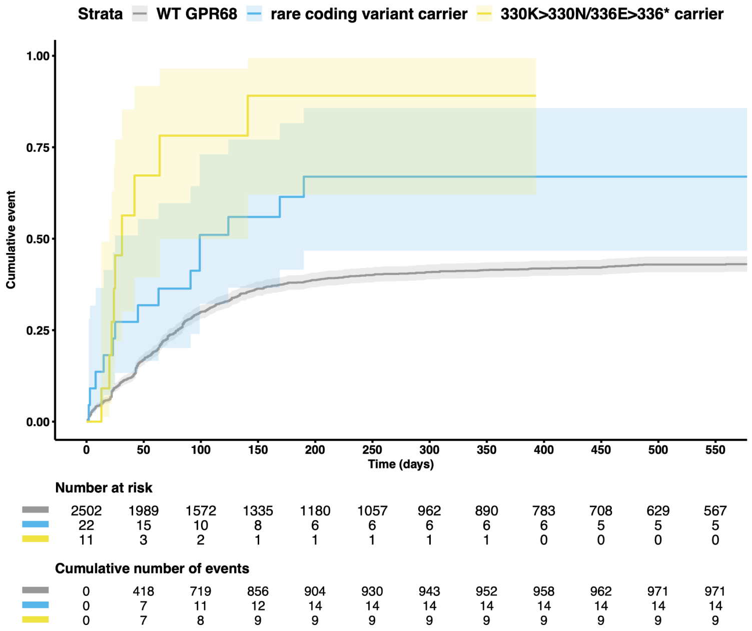


**Figure S5.** Cumulative incidence plot for PN events in taxane treated patients stratified by rare coding variant burden in *GPR68*.

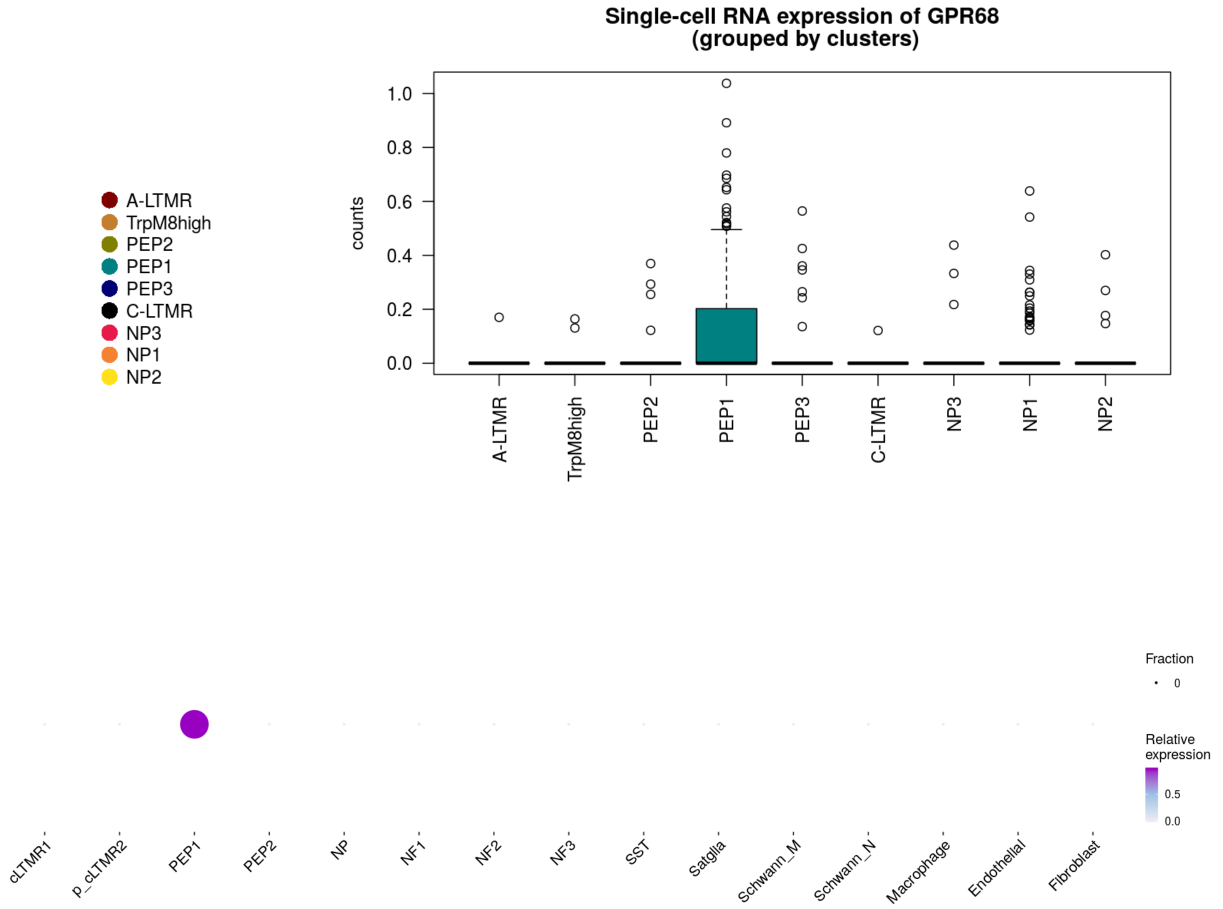


**Figure S6.** (top) Relative expression patterns *GPR68* in different DRG cell types in macaque (top from https://ernforsgroup.shinyapps.io/macaquedrg/) [2] expressed in normalized in mouse as illustrated by boxplots. (bottom from https://painseq.shinyapps.io/publish/#)[3] indicates a similar expression pattern to *GPR68* in human DRG. PEP1 neurons show relatively high expression of *GRP68* in both organisms illustrated by relative expression dot plot.

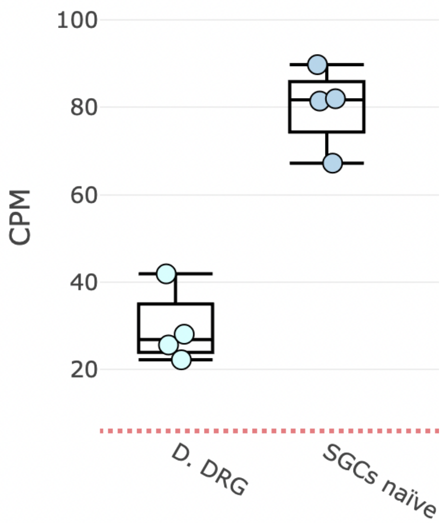


**Figure S7**. Normalized counts per million expression of *GRID2* in bulk RNA-seq from human DRG (D. DRG) as compared to sorted satellite glial cells (SCGCs) in mice (http://rna-seq-browser.herokuapp.com/)[4].

| | | | Sex | | Age | | | PN Event Grade | | | | | |
|---|---|---|---|---|---|---|---|---|---|---|---|---|---|
| **Trial** | **Arm** | **Chemo Naive** | **M** | **F** | **≤65** | **>65** | **Diab** | **None** | **1, n** | **%** | **>1,n** | **%** | **N (all)** |
| ido | Aido | N | 25 | 48 | 48 | 25 | 9 | 69 | 2 | 3 | 2 | 3 | 73 |
| ima050 | APCB | Y | 0 | 317 | 213 | 104 | 33 | 132 | 140 | 44 | 45 | 14 | 317 |
| ima050 | PCB | Y | 0 | 328 | 220 | 108 | 33 | 145 | 130 | 40 | 53 | 16 | 328 |
| imm151 | AB | Y | 140 | 67 | 130 | 77 | 39 | 194 | 10 | 5 | 3 | 1 | 207 |
| imm151 | SUN | Y | 147 | 42 | 132 | 57 | 31 | 176 | 12 | 6 | 1 | 1 | 189 |
| imp110 | Atezo | Y | 59 | 35 | 47 | 47 | 5 | 91 | 1 | 1 | 2 | 2 | 94 |
| imp110 | Chemo | Y | 55 | 24 | 38 | 41 | 12 | 77 | 1 | 1 | 1 | 1 | 79 |
| imp130 | ACNabP | Y | 110 | 89 | 114 | 85 | 34 | 143 | 35 | 18 | 21 | 11 | 199 |
| imp130 | CNabP | Y | 55 | 46 | 49 | 52 | 18 | 77 | 10 | 10 | 14 | 14 | 101 |
| imp131 | ACNabP | Y | 125 | 33 | 89 | 69 | 38 | 111 | 31 | 20 | 16 | 10 | 158 |
| imp131 | ACP | Y | 131 | 33 | 80 | 84 | 32 | 91 | 42 | 26 | 31 | 19 | 164 |
| imp131 | CNabP | Y | 125 | 32 | 86 | 71 | 34 | 121 | 18 | 11 | 18 | 11 | 157 |
| imp132 | ACPem | Y | 80 | 43 | 79 | 44 | 20 | 114 | 5 | 4 | 4 | 3 | 123 |
| imp132 | CPem | Y | 77 | 30 | 67 | 40 | 16 | 102 | 4 | 4 | 1 | 1 | 107 |
| imp133 | ACE | Y | 52 | 25 | 51 | 26 | 19 | 71 | 3 | 4 | 3 | 4 | 77 |
| imp133 | CE | Y | 40 | 32 | 43 | 29 | 13 | 69 | 3 | 4 | 0 | 0 | 72 |
| imp150 | ABCP | Y | 117 | 87 | 122 | 82 | 26 | 109 | 61 | 30 | 34 | 17 | 204 |
| imp150 | ACP | Y | 131 | 96 | 140 | 87 | 25 | 132 | 58 | 26 | 37 | 16 | 227 |
| imp150 | BCP | Y | 105 | 75 | 111 | 69 | 29 | 107 | 41 | 23 | 32 | 18 | 180 |
| impas130 | ANabP | Y | 1 | 155 | 118 | 38 | 14 | 112 | 16 | 10 | 28 | 18 | 156 |
| impas130 | NabP | Y | 1 | 136 | 101 | 36 | 11 | 93 | 26 | 19 | 18 | 13 | 137 |
| ims170 | Acobi | Y | 86 | 56 | 69 | 73 | 17 | 136 | 2 | 1 | 4 | 3 | 142 |
| ims170 | Apembro | Y | 82 | 49 | 70 | 61 | 19 | 129 | 1 | 1 | 1 | 1 | 131 |
| imv010 | Atezo | N | 148 | 37 | 88 | 97 | 26 | 183 | 1 | 1 | 1 | 1 | 185 |
| imv010 | Observ | N | 150 | 37 | 86 | 101 | 31 | 181 | 4 | 2 | 2 | 1 | 187 |
| imv130 | AGC | Y | 135 | 33 | 73 | 95 | 35 | 148 | 14 | 8 | 6 | 4 | 168 |
| imv130 | Atezo | Y | 100 | 27 | 52 | 75 | 20 | 124 | 1 | 1 | 2 | 2 | 127 |
| imv130 | GC | Y | 132 | 53 | 56 | 129 | 40 | 180 | 3 | 2 | 2 | 1 | 185 |
| imv211 | Atezo | N | 170 | 49 | 98 | 121 | 29 | 206 | 8 | 4 | 5 | 2 | 219 |
| imv211 | Chemo | N* | 167 | 40 | 80 | 127 | 35 | 160 | 26 | 13 | 21 | 10 | 207 |

**Table S1.** Frequency and grade of PN events in cancer patients of European ancestry across 14 randomized controlled trials testing immunotherapy and chemotherapy combinations. Chemo naive designates that patients in the trial arm did not receive any prior chemotherapy. N* designates patients that were taxane naive. Diab column indicates the number of patients in the trial arm that had a prior diabetes diagnosis before treatment for the given trial arm. Trial abbreviations are as follows: impXXX = IMpowerXXX, imm151=IMmotion151, imvXX = IMvigorXXX; ims170=IMspire170; imvXXX=IMvigorXXX; impas130=IMpassion130. Treatments in the trial arms are abbreviated as follows: A = atezolizumab, ido = ido inhibitor; Atezo = atezolizumab monotherapy; B = bevacizumab; cobi=cobimetinib; Pembro=pembrolizumab; C = carboplatin or cisplatin ; P = paclitaxel; NabP = nab-paclitaxel; Pem=Pemetrexed; G=Gemcitabine; Observ = designates observation after surgery; Chemo = physicians choice chemotherapy vinflunine, docetaxel, or paclitaxel in IMvigor211; and carboplatin or cisplatin plus pemetrexed or gemcitabine in IMpower110; E = etoposide; SUN = sunitinib. Red rows designate where both a taxane and platinum-based chemotherapy was used. Yellow rows designate use of only platinum-based chemotherapy. Blue rows designate use of only a taxane chemotherapy. Chemotherapy regimens are provided in Tables S2-S3.

| Trial | Arm Abbrev. | Taxane Dosing |
|---|---|---|
| ima050 | APCPB and PCB | Paclitaxel 175 mg/$m^2$ on Day 1 of each 21-day cycle |
| imp130 | ACNabP, CNabP | Nab-paclitaxel at 100 mg/$m^2$ on Days 1, 8, and 15 of each 21-day cycle. |
| imp131 | ACNabP, CNabP | Nab-paclitaxel 100 mg/$m^2$ on Day 1, 8, and 15 of each 21-day cycle |
| imp150 | ABCP, ACP, BCP | Paclitaxel at 200 mg/$m^2$ on Day 1 of each 21-day cycle |
| impas130 | ANabP, NabP | Nab-Paclitaxel at 100 mg/$m^2$ on Days 1, 8, and 15 of each 28-day cycle |
| imv211 | Chemo | Docetaxel or paclitaxel 75 mg/$m^2$ on Day 1 of each 21-day cycle |

**Table S2 - Taxane Regimens**

| Trial | Arm Abbrev. | Platinum Chemotherapy Dosing |
|---|---|---|
| ima050 | APCPB and PCB | ˙Carboplatin at AUC of 6 mg/mL min on Day 1 of each 21-day cycle |
| imp110 | Chemo | ˙Carboplatin at AUC 6 when given in combination with pemetrexed or at a dose of AUC 5 when given in combination with gemcitabine, every 21 days.<br>˙Cisplatin at 75 mg/$m^2$ every 21 days. |
| imp130 | ACNabP, CNabP | ˙Carboplatin was administered at AUC 6 mg/mL/min on Day 1 of each 21-day cycle |
| imp131 | ACNabP, ACP, CNabP | ˙Carboplatin AUC 6 mg/mL/min on Day 1 of each 21-day cycle |
| imp132 | ACPem, CPem | ˙Carboplatin on Day 1 q3w for 4 or 6 cycles (Cycle length=21 days) in induction dosing period with doses calculated using Calvart formula.<br>˙Cisplatin at 75 mg/$m^2$ with cycle length=21 days |
| **imp133** | ACE, CE | ˙Carboplatin intravenous infusion to achieve an initial target AUC of 5 mg/mL/min was administered on Day 1 of each 21-day cycle. |
| **imp150** | ABCP, ACP, BCP | ˙Carboplatin was administered at AUC 6 mg/mL/min on Day 1 of each 21-day cycle |
| **imv130** | | ˙Carboplatin to AUC of 4.5 mg/mL min by IV infusion on Day 1 of each 21-day cycle. ˙Cisplatin will be administered at a dose of 70 mg/$m^2$ by IV infusion on Day 1 of each 21-day cycle. |

**Table S3 - Platinum Chemotherapy Regimens**

N=4,900 all patients

| Index Variant | Alleles | Effect AF | HR [95% CI] | p | Nearest Gene | Location |
|---|---|---|---|---|---|---|
| rs17020773 | T/C | 0.03 | 1.86[1.50-2.31] | $2.03 \cdot 10^{-8}$ | *GRID2* | intronic |
| rs115575220 | G/T | 0.01 | 2.44[1.77-3.35] | $4.15 \cdot 10^{-8}$ | *SCG2* | intergenic (126kb) |
| rs191482247 | A/G | 0.01 | 2.04[1.52-2.73] | $1.40 \cdot 10^{-6}$ | *ZDHHC14* | intronic |

N=2,535 taxane treated subcohort

| Index Variant | Alleles | Effect AF | HR [95% CI] | p | Nearest Gene | Location |
|---|---|---|---|---|---|---|
| rs17020773 | T/C | 0.03 | 1.96[1.56-2.47] | $6.36 \cdot 10^{-9}$ | *GRID2* | intronic |
| rs191482247 | A/G | 0.01 | 2.29[1.71-3.06] | $2.54 \cdot 10^{-8}$ | *ZDHHC14* | intronic |
| rs115575220 | G/T | 0.01 | 2.43[1.72-3.43] | $4.5 \cdot 10^{-7}$ | *SCG2* | intergenic (126kb) |

**Table S4.** Low frequency variants that were associated with risk of PN at $p < 5 \cdot 10^{-8}$ across N=4,900 cancer patients or within the N=2,535 taxane treated cohort. AF = allele frequency. Alleles are designated as: (non-effect)/(affect allele). Hazard ratio (HR) is expressed in dosage of the effect allele.

| rsid | Candidate Gene | p (all cohort) | p (taxane) |
|---|---|---|---|
| rs10486003 | TAC1 | 0.82 | 0.55 |
| rs10771973 | FGD4 | 0.82 | 0.47 |
| rs2228001 | XPC | 0.90 | 0.58 |
| rs2302237 | SCN4A | 0.94 | 0.90 |
| rs3114018 | ABCG2 | 0.16 | 0.19 |
| **rs3125923** | **GPR177** | **0.03** | **0.05** |
| rs3213619 | ABCB1 | 0.90 | 0.75 |
| rs3213619 | ABCB1 | 0.90 | 0.75 |
| rs3213619 | ABCB1 | 0.90 | 0.75 |
| rs3213619 | ABCB1 | 0.90 | 0.75 |
| rs4737264 | XKR4 | 0.63 | 0.28 |
| rs6438552 | GSK3B | 0.13 | 0.05 |
| rs6746030 | SCN9A | 0.64 | 0.75 |
| **rs7001034** | **FZD3** | **0.04** | **0.16** |
| rs7349683 | EPHA5 | 0.58 | 0.71 |
| rs74497159 | S1PR1 | 0.87 | 0.49 |
| rs875858 | VAC14 | 0.89 | 0.59 |
| rs9501929 | TUBB2A | 0.12 | 0.33 |
| rs9937 | RRM1 | 0.55 | 0.56 |

**Table S5 - Replication of Variants Previously Associated with CIPN.**

**References**

1. Bakken TE, Jorstad NL, Hu Q, Lake BB, Tian W, Kalmbach BE, et al. Comparative cellular analysis of motor cortex in human, marmoset and mouse. Nature. 2021;598:111–9.
2. Kupari J, Usoskin D, Parisien M, Lou D, Hu Y, Fatt M, et al. Single cell transcriptomics of primate sensory neurons identifies cell types associated with chronic pain. Nat Commun. 2021;12:1510.
3. Renthal W, Tochitsky I, Yang L, Cheng Y-C, Li E, Kawaguchi R, et al. Transcriptional Reprogramming of Distinct Peripheral Sensory Neuron Subtypes after Axonal Injury. Neuron. 2020;108:128-144.e9.
4. Liang Z, Hore Z, Harley P, Stanley FU, Michrowska A, Dahiya M, et al. A transcriptional toolbox for exploring peripheral neuroimmune interactions. Pain. 2020;161:2089–106.
